# Statin use, survival and incidence of thrombosis among older patients with polycythemia vera and essential thrombocythemia

**DOI:** 10.1002/cam4.6528

**Published:** 2023-09-13

**Authors:** Nikolai A. Podoltsev, Rong Wang, Rory M. Shallis, Jessica M. Stempel, Mengyang Di, Natalia Neparidze, Amer M. Zeidan, Scott F. Huntington, Smith Giri, Sarah C. Hull, Steven D. Gore, Xiaomei Ma

**Affiliations:** ^1^ Section of Hematology, Department of Internal Medicine Yale School of Medicine New Haven Connecticut USA; ^2^ Cancer Outcomes, Public Policy, and Effectiveness Research (COPPER) Center Yale University New Haven Connecticut USA; ^3^ Department of Chronic Disease Epidemiology Yale School of Public Health New Haven Connecticut USA; ^4^ Section of Cardiology, Department of Internal Medicine Yale School of Medicine New Haven Connecticut USA; ^5^ Present address: Division of Hematology and Oncology University of Alabama School of Medicine Birmingham Alabama USA; ^6^ Present address: Investigational Drug Branch, Cancer Therapy Evaluation Program National Cancer Institute Bethesda Maryland USA

**Keywords:** MPN, statins, survival, thrombosis

## Abstract

**Background:**

Polycythemia vera (PV) and essential thrombocythemia (ET) are linked to increased risk of cardiovascular morbidity and mortality. In addition to the reduction in of arterial thrombotic events, statins may prevent venous thrombosis including among patients with cancer. As previous registry‐ and claims‐based studies revealed that the use of statins may improve the survival of patients with various malignancies we evaluated their impact on outcomes of older adults with PV and ET.

**Methods:**

We identified 4010 older adults (aged 66–99 years at diagnosis) with PV (*n* = 1809) and ET (*n* = 2201) in a population‐based cohort study using the Surveillance, Epidemiology, and End Results‐Medicare database with median follow‐up of 3.92 (interquartile range: 2.58–5.75) years. Propensity score matching (PSM) and inverse probability of treatment weighting (IPTW) approaches were utilized to assess potential association between statins and overall survival. Multivariable competing risk models with death as a competing risk were used to evaluate possible relationship between statins and the incidence of thrombosis.

**Results:**

55.8% of the patients used statins within the first year after PV/ET diagnosis, and statin use was associated with a 22% reduction in all‐cause mortality (PSM: hazard ratio [HR] = 0.78, 95% confidence interval [CI]: 0.63–0.98, *p* = 0.03; IPTW: HR = 0.79, 95% CI: 0.64–0.97, *p* = 0.03). Statins also reduced the risk of thrombosis in this patient population (PSM: HR = 0.63, 95% CI: 0.51–0.78, *p* < 0.01; IPTW: HR = 0.57, 95% CI: 0.49–0.66, *p* < 0.01) as well as in PV and ET subgroups.

**Conclusions:**

These findings suggest that it may be important to incorporate statins into the therapeutic strategy for older adults with PV and ET.

## 1 INTRODUCTION

Polycythemia vera (PV) and essential thrombocythemia (ET), two types of Philadelphia chromosome‐negative myeloproliferative neoplasms (MPNs), are closely related clonal disorders with driver mutations leading to Janus kinase/signal transducers and activators of transcription (*JAK–STAT*) pathway activation. The most common mutation, *JAK2‐V617F*, is observed in over 95% of patients with PV and in about 60% of patients with ET.^1^ Additionally, *JAK2* mutations and activation of the *JAK–STAT* pathway are implicated in the chronic inflammatory state associated with MPN progression, development of second lymphoid and solid malignancies, and increased cardiovascular disease risk.^2^, ^3^ Patients with PV and ET face elevated risks of arterial and venous thrombotic events with higher rates observed in patients with PV.^1^ Arterial thromboses are more common than venous events in MPNs with the highest rates around the time of diagnosis which decrease over time, likely due to the effects of treatment. Important risk factors for these events include age and previous thrombotic episodes.^4^ As cardiovascular mortality is one of the major causes of death among PV and ET patients,^5^ reducing the risk of thrombotic events is the primary goal of treatment for PV and ET, accomplished by therapeutic phlebotomies in PV and aspirin as well as cytoreductive therapies in both PV and ET. Consensus MPN clinical guidelines recommend addressing modifiable cardiovascular risk factors in patients with PV and ET.^6^, ^7^

Statins (3‐hydroxy‐3‐methylglutaryl‐coenzyme A reductase inhibitors), a group of lipid‐lowering drugs, are commonly used for primary and secondary prevention of atherosclerotic cardiovascular disease based on evidence that they stabilize atherosclerotic plaque via lipid‐lowering and anti‐inflammatory effects and ultimately reduce both cardiovascular events and mortality.^8^, ^9^ In addition to the reduction of arterial thrombotic events, statins have venous antithrombotic effects^10^ and are effective as primary and secondary prevention of venous thromboembolism,^11^ including among patients with cancer.^12^ These effects may be in part due to suppression of platelet function.^13^ Furthermore, statins inhibit cell proliferation, promote apoptosis and tumor cell differentiation, and modulate the tumor microenvironment, all qualities that invoke the possibility of statins possessing anticancer properties.^14^, ^15^ Indeed, previous registry‐ and claims‐based studies have shown that the use of statins improved the survival of patients with various solid tumors,^16^, ^17^, ^18^, ^19^, ^20^, ^21^ including several meta‐analyses.^22^, ^23^, ^24^, ^25^

Statins were recently shown to exert a protective effect on the development of MPNs in a large population‐based cohort study,^26^ and various reports have suggested that statins may be effective as a potential therapeutic approach for MPNs.^27^, ^28^, ^29^, ^30^ The use of statins in PV led to a reduction in the number of phlebotomies in a retrospective multicenter study suggesting the potential of statins to decrease *JAK2*‐dependent cellular proliferation.^31^

To better understand the impact of statin use on MPN patients' survival and thrombotic risk after MPN diagnosis, we conducted a large population‐based cohort study of older adults diagnosed with PV or ET in the United States, with extended follow‐up.

## 2 METHODS

**FIGURE 1:**
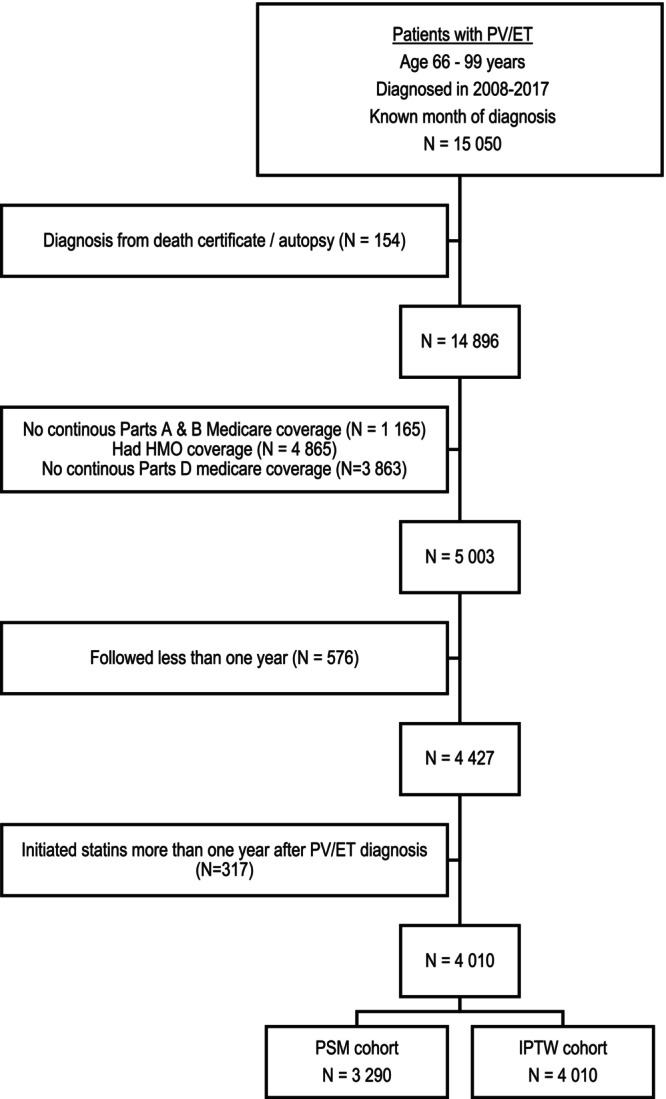
Selection of Study Population. ET, essential thrombocythemia; HMO, health maintenance organization; IPTW, inverse probability of treatment weighting; PSM, propensity score matching; PV, polycythemia vera.

Using the Surveillance, Epidemiology, and End Results (SEER)‐Medicare database, we enrolled patients with PV (International Classification of Diseases for Oncology, third edition [ICD‐O‐3] 9950) and ET (ICD‐O‐39962) who were diagnosed in 2008–2017, were aged 66–99 years at diagnosis, had continuous Medicare Parts A, B, and D coverage, but not enrolled in health maintenance organizations from 12 months before diagnosis to the end of follow‐up (i.e., death, the end of study on December 31, 2019 or changed insurance status, whichever was earlier), had been followed for ≥1 year after diagnosis, but not reported from death certificate or autopsy only (Figure 1). The Yale Human Investigation Committee determined that this study did not directly involve human subjects.

We searched Part D claims for statins (rosuvastatin, atorvastatin, pitavastatin, simvastatin, lovastatin, pravastatin, or fluvastatin) prescriptions. The primary outcome was overall survival. To reduce immortal time bias, we limited statin users to those who received statins within the first year after MPN diagnosis and excluded patients with delayed statin initiation. We obtained information on age at diagnosis, sex, race/ethnicity, marital status, state buy‐in, census tract Yost index (a composite socioeconomic status index),^32^ disability status,^33^ Elixhauser comorbidity score,^34^ history of thrombosis, influenza vaccination, and hydroxyurea use. For PV patients, we also assessed therapeutic phlebotomy use.

To address the potential for confounding by statin use, we evaluated each patient's likelihood of being a post‐diagnosis statin user via a logistic regression model. As shown in Figure 1, we created two separate cohorts. One was the 1:1 nearest‐neighbor propensity score matching (PSM) without replacement cohort. The other was the inverse probability treatment weighting (IPTW) cohort, for which we estimated treatment weights for each participant, proportional to the inverse probability of statin use. For both cohorts, standardized differences were calculated to assess the balance achieved between the treatment groups by the matching/weighting process. Covariates with standardized differences above 0.10 were also incorporated in the final regression models to reduce any residual selection bias.

Time to event was analyzed with weighted Kaplan–Meier methods and log‐rank test. Multivariable Cox proportional hazards models that included a variable for statin use before MPN diagnosis and imbalanced variables were used to estimate hazards ratios (HRs) and 95% confidence intervals (CIs). For the IPTW cohort, the Cox model was also weighted by the IPTW. Our secondary outcome of interest was first incident thrombotic events. The cumulative incidence function of thrombosis was computed via a competing risk model. Comparisons of cumulative incidence across treatment groups were performed using Gray's test.^35^ Multivariable competing risk models were fitted^36^ to evaluate the relationship between statin use and risk of thrombosis after MPN diagnosis. Death was considered a competing event. We also conducted analyses for each MPN subtype. To remove potential influence of statins taken prior to MPN diagnosis on the study outcomes, sensitivity analyses excluded this group of statin users.

All tests were two‐sided with an alpha of 0.05 and were conducted in SAS Version 9.4 (SAS Inc.).

## 3 RESULTS

The final cohort included 4010 patients (1809 PV and 2201 ET). The majority of patients were female (63.0%) and white (86.8%). The median age at diagnosis was 77 years for both patients with PV (IQR: 71–82) and ET (IQR: 72–84). 55.8% of patients (1011 PV and 1228 ET) used statins within the first year after MPN diagnosis with an 88.7% (IQR: 64.9%–97.5%) days covered by statins since initiation. Patients who received statins during the first year after MPN diagnosis were younger, more likely to be male, had more comorbidities, were more likely to have a history of thrombosis and received influenza vaccination in the year before MPN diagnosis than those who did not receive statins (all *p* < 0.01, Table 1). After weighting by inverse probability of treatment, no difference was observed between statin users and nonusers. However, in the PSM cohort, which included 1645 matched pairs, statin users were still less likely to be white (85.3% vs. 88.3%), more likely to have more comorbidities (46.7% vs. 41.9%) and a history of thrombosis (19.2% vs. 14.3%) than their matched counterparts (Table 1).

**TABLE 1:** Characteristics of 4010 Patients with myeloproliferative neoplasms, 2008–2017.

Statin use	Pre‐matching/weighting cohort				Propensity score matching			Weighted by IPTW		
	User	Nonuser			User	Nonuser		User	Nonuser	
	*N* (%)	*N* (%)	P^a^	%Std. diff	*N* (%)	*N* (%)	%Std. diff	(Weighted %)	(Weighted %)	%Std. diff
Subtype										
PV	1011 (45.2)	798 (45.1)	0.95	0.19	747 (45.4)	746 (45.3)	0.12	(45.3)	(45.3)	0.11
ET	1228 (54.8)	973 (54.9)		−0.19	898 (54.6)	899 (54.7)	−0.12	(54.7)	(54.7)	−0.11
Age at diagnosis (years)										
66–69	354 (15.8)	290 (16.4)	<0.01	−1.54	277 (16.8)	265 (16.1)	1.97	(16.0)	(16.0)	0.04
70–74	525 (23.4)	387 (21.9)		3.81	370 (22.5)	356 (21.6)	2.05	(22.9)	(23.0)	−0.32
75–79	519 (23.2)	370 (20.9)		5.52	352 (21.4)	341 (20.7)	1.64	(21.8)	(21.6)	0.36
80–84	453 (20.2)	340 (19.2)		2.60	321 (19.5)	325 (19.8)	−0.61	(20.0)	(20.2)	−0.42
85–99	388 (17.3)	384 (21.7)		−11.0	325 (19.8)	358 (21.8)	−4.95	(19.4)	(19.2)	0.36
Sex										
Female	1354 (60.5)	1173 (66.2)	<0.01	−12.0	1030 (62.6)	1068 (64.9)	−4.81	(62.8)	(62.3)	1.19
Male	885 (39.5)	598 (33.8)		11.98	615 (37.4)	577 (35.1)	4.81	(37.2)	(37.7)	−1.19
Race										
White	1908 (85.2)	1573 (88.8)	<0.01	−10.7	1404 (85.3)	1452 (88.3)	−8.63	(86.8)	(86.8)	0.08
Other	331 (14.8)	198 (11.2)		10.74	241 (14.7)	193 (11.7)	8.63	(13.2)	(13.2)	−0.08
Marital status										
Married	738 (33.0)	565 (31.9)	0.60	2.26	534 (32.5)	527 (32.0)	0.91	(32.5)	(32.7)	−0.51
Unmarried	1327 (59.3)	1054 (59.5)		−0.50	978 (59.5)	979 (59.5)	−0.12	(59.5)	(59.3)	0.36
Unknown	174 (7.8)	152 (8.6)		−2.96	133 (8.1)	139 (8.4)	−1.32	(8.0)	(8.0)	0.22
Hydroxyurea use										
No	550 (24.6)	479 (27.0)	0.07	−5.68	426 (25.9)	430 (26.1)	−0.55	(25.6)	(25.4)	0.48
Yes	1689 (75.4)	1292 (73.0)		5.68	1219 (74.1)	1215 (73.9)	0.55	(74.4)	(74.6)	−0.48
Elixhauser comorbidity score^b^										
0	189 (8.4)	332 (18.7)	<0.01	−30.4	189 (11.5)	216 (13.1)	−5.00	(13.0)	(13.0)	0.25
1	881 (39.3)	750 (42.3)		−6.11	687 (41.8)	740 (45.0)	−6.50	(40.6)	(40.5)	0.12
≥2	1169 (52.2)	689 (38.9)		26.96	769 (46.7)	689 (41.9)	9.80	(46.4)	(46.5)	−0.29
History of thrombosis										
No	1718 (76.7)	1536 (86.7)	<0.01	−26.1	1329 (80.8)	1410 (85.7)	−13.2	(81.1)	(80.8)	0.60
Yes	521 (23.3)	235 (13.3)		26.10	316 (19.2)	235 (14.3)	13.22	(18.9)	(19.2)	−0.60
Disability status										
No	1985 (88.7)	1573 (88.8)	0.87	−0.52	1455 (88.4)	1461 (88.8)	−1.15	(88.8)	(88.9)	−0.14
Yes	254 (11.3)	198 (11.2)		0.52	190 (11.6)	184 (11.2)	1.15	(11.2)	(11.1)	0.14
State buy‐in										
No	1823 (81.4)	1467 (82.8)	0.25	−3.69	1342 (81.6)	1362 (82.8)	−3.18	(82.2)	(82.1)	0.34
Yes	416 (18.6)	304 (17.2)		3.69	303 (18.4)	283 (17.2)	3.18	(17.8)	(17.9)	−0.34
Yost index										
Fifth quintile (highest SES)	708 (31.6)	586 (33.1)	0.23	−3.14	473 (28.8)	534 (32.5)	−8.05	(32.2)	(32.2)	0.04
Fourth quintile	445 (19.9)	382 (21.6)		−4.18	376 (22.9)	355 (21.6)	3.07	(20.8)	(21.0)	−0.35
Third quintile	402 (18.0)	281 (15.9)		5.57	291 (17.7)	266 (16.2)	4.05	(17.0)	(16.8)	0.56
Second quintile	354 (15.8)	288 (16.3)		−1.23	275 (16.7)	263 (16.0)	1.97	(16.1)	(16.1)	−0.11
First quintile (lowest SES)	244 (10.9)	175 (9.9)		3.33	174 (10.6)	168 (10.2)	1.20	(10.4)	(10.4)	−0.03
Unknown	86 (3.8)	59 (3.3)		2.74	56 (3.4)	59 (3.6)	−0.99	(3.5)	(3.6)	−0.22
Receipt of influenza vaccination in the 12 months before PV or ET diagnosis										
No	760 (33.9)	712 (40.2)	<0.01	−13.0	625 (38.0)	635 (38.6)	−1.25	(36.9)	(36.8)	0.23
Yes	1479 (66.1)	1059 (59.8)		12.99	1020 (62.0)	1010 (61.4)	1.25	(63.1)	(63.2)	−0.23

Abbreviations: CI, confidence interval; ET, essential thrombocythemia; IPTW, inverse probability of treatment weighting; PV, polycythemia vera; Std. diff, standardized difference.

^a^ *p*‐Values were derived from chi‐squared tests for categorical variables and *t*‐tests for continuous variables.

^b^ Prior thrombotic events were not included in the modified Elixhauser score.

### 3.1 Statin use and overall survival

**FIGURE 2:**
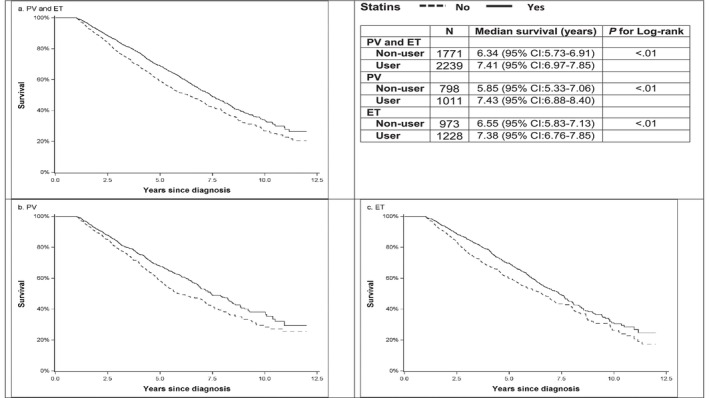
(a) Overall survival by statin use among patients with ET and PV. (b) Overall survival by statin use among patients with PV. (c) Overall survival by statin use among patients with ET.

After a median follow‐up of 3.92 (IQR: 2.58–5.75) years, 35.4% (*n* = 792) of statin users and 41.8% (*n* = 741) of nonusers died. Statin users had a significantly better overall survival than nonusers (Log‐rank test, *p* < 0.01) (Figure 2A). In the Cox proportional hazards model, statin use was associated with a 22% reduction in the risk of all‐cause mortality in the PSM cohort (hazard ratio [HR] = 0.78, 95% confidence interval [CI]: 0.63–0.98, *p* = 0.03) and a 21% reduction in the IPTW cohort (HR = 0.79, 95% CI: 0.64–0.97, *p* = 0.03) (Table 2).

**TABLE 2:** Multivariable Cox proportional hazards analysis for overall survival among PV and ET patients.

	Propensity score matching				Inverse probability of treatment weighting			
Statin use	*N*	HR	95% CI	*p*	*N*	HR	95% CI	*p*
Primary analysis								
*Overall*								
Never	1645	1.00			1771	1.00		
Ever	1645	0.78^a^	0.63–0.98	0.03	2239	0.79^b^	0.64–0.97	0.03
*PV*								
Never	727	1.00			798	1.00		
Ever	727	0.94^c^	0.69–1.29	0.71	1011	0.82^b^	0.59–1.14	0.24
*ET*								
Never	888	1.00			973	1.00		
Ever	888	0.79^c^	0.60–1.05	0.10	1228	0.77^b^	0.59–1.02	0.07
Sensitivity analysis								
*Overall*								
Never	582	1.00			1708	1.00		
Ever	582	0.59^d^	0.50–0.71	<0.01	584	0.66	0.57–0.77	<0.01
*PV*								
Never	267	1.00			772	1.00		
Ever	267	0.68^e^	0.52–0.90	0.01	269	0.76^f^	0.61–0.96	0.02
*ET*								
Never	314	1.00			936	1.00		
Ever	314	0.60^g^	0.47–0.76	<0.01	315	0.61^h^	0.50–0.76	<0.01

Abbreviations: CI, confidence interval; ET, essential thrombocythemia; HR, hazard ratio; N, number of patients; PV, polycythemia vera.

^a^ Adjusted for use of statins before diagnosis and history of thrombosis in the model.

^b^ Adjusted for use of statins before diagnosis.

^c^ Adjusted for use of statins before diagnosis, history of thrombosis and Elixhauser comorbidity score in the model.

^d^ Adjusted for age at diagnosis, Yost index in the model.

^e^ Adjusted for history of thrombosis in the model.

^f^ Adjusted for receipt of influenza vaccination in the 12 months before diagnosis in the model.

^g^ Adjusted for race and disability status in the model.

^h^ Adjusted for age at diagnosis in the model.

Among patients with PV, 35.0% (*n* = 354) of statin users and 43.0% (*n* = 343) of nonusers died after a median follow up of 4.00 years. Statin users had a significantly better overall survival than nonusers (Log‐rank test, *p* < 0.01) (Figure 2B). In the Cox model, statins use was not associated with the risk of all‐cause mortality in this subgroup of patients (PSM cohort: HR = 0.94, 95% CI: 0.69–1.29, *p* = 0.71; IPTW cohort: HR = 0.82, 95% CI: 0.59–1.14, *p* = 0.24) (Table 2).

Among patients with ET, 35.7% (*n* = 438) of statin users and 40.9% (*n* = 398) of nonusers died after a median follow‐up of 3.84 years. As shown in Figure 2C, patients with ET who used statins had better survival than nonusers. After taking confounders into consideration, there was no difference in overall survival between statin users and nonusers (PSM cohort: HR = 0.79, 95% CI: 0.60–1.05, *p* = 0.10; IPTW cohort: HR = 0.77, 95% CI: 0.59–1.02, *p* = 0.07) among patients with ET (Table 2).

We conducted a sensitivity analysis by excluding 2135 patients who received statins before MPN diagnosis. After the exclusion, 2292 (1041 PV and 1251 ET) patients remained. As shown in Table 2, statin use was associated with better overall survival among all patients as well as among patients in the PV and ET subgroups.

### 3.2 Statin use and thrombosis risk

**FIGURE 3:**
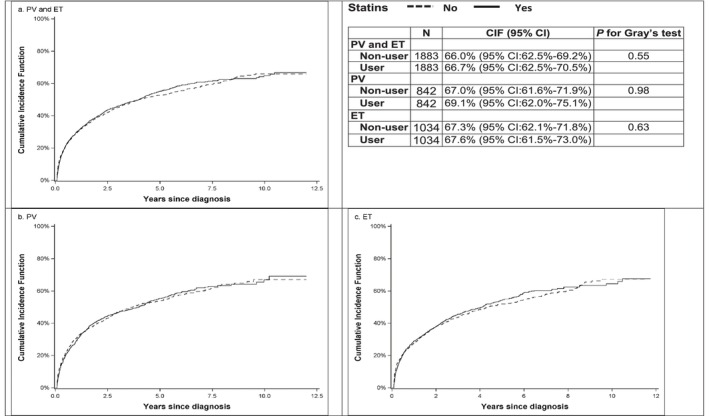
(a) Cumulative incidence function for thrombotic events by statin use among patients with PV and ET. (b) Cumulative incidence function for thrombotic events by statin use among patients with PV. (c) Cumulative incidence function for thrombotic events by statin use among patients with ET.

Thrombosis after diagnosis was observed in 2243 (52.7%; 1022 PV and 1221 ET) patients. Among these thrombotic events, 1804 (80.4%; 791 PV and 1013 ET) were arterial thromboses. Of all 4010 patients with PV and ET, 1129 (53.5%) statin users and 1114 (51.8%) nonusers had thrombosis after diagnosis. Although cumulative incidence function curves showed no difference in thrombosis occurrence between statins users and nonusers (Figure 3), in the Cox models, statin use reduced the risk of thrombosis by nearly 40% (PSM: HR = 0.63, 95% CI: 0.51–0.78, *p* < 0.01; IPTW: HR = 0.57, 95% CI: 0.49–0.66, *p* < 0.01) (Table 3). In addition, decreased risk of thrombosis was also observed in patients with PV (PSM: HR = 0.60, 95% CI: 0.45–0.81, *p* < 0.01; IPTW: HR = 0.58, 95% CI: 0.85–0.71, *p* < 0.01) (Table 3) and patients with ET (PSM: HR = 0.63, 95% CI: 0.46–0.85, *p* < 0.01; IPTW: HR = 0.57, 95% CI: 0.46–0.70, *p* < 0.01) (Table 3) who used the statins. Sensitivity analysis, which excluded patients who used statins prior to MPN diagnosis, showed similar results (Table 3).

**TABLE 3:** Multivariable competing risk models for thrombosis among PV and ET patients.

	Propensity score matching				Inverse probability treatment weighting			
Statin use	*N*	HR	95% CI	*p*	*N*	HR	95% CI	*p*
Primary analysis								
*Overall*								
Never	1883	1.00			2150	1.00		
Ever	1883	0.63^a^	0.51–0.78	<0.01	2110	0.57^b^	0.49–0.66	<0.01
*PV*								
Never	842	1.00			965	1.00		
Ever	842	0.60 ^b^	0.45–0.81	<0.01	948	0.58 ^b^	0.48–0.71	<0.01
*ET*								
Never	1034	1.00			1185	1.00		
Ever	1034	0.63 ^b^	0.46–0.85	<0.01	1162	0.57 ^b^	0.46–0.70	<0.01
Sensitivity analysis								
*Overall*								
Never	273	1.00			2019	1.00		
Ever	273	0.58^c^	0.45–0.74	<0.01	273	0.69	0.64–0.75	<0.01
*PV*								
Never	135	1.00			906	1.00		
Ever	135	0.65^d^	0.47–0.90	<0.01	135	0.70^e^	0.62–0.79	<0.01
*ET*								
Never	138	1.00			1113	1.00		
Ever	138	0.65^f^	0.44–0.95	0.03	138	0.66^g^	0.58–0.74	<0.01

Abbreviations: CI, confidence interval; ET, essential thrombocythemia; HR, hazard ratio; N, number of patients; PV, polycythemia vera.

^a^ Adjusted for use of statins before diagnosis and history of thrombosis in the model.

^b^ Adjusted for use of statins before diagnosis.

^c^ Adjusted for age at diagnosis in the model.

^d^ Adjusted for age at diagnosis, Elixhauser comorbidity score, disability status, and Yost index in the model.

^e^ Adjusted for age at diagnosis, marital status, and Elixhauser comorbidity score in the model.

^f^ Adjusted for race, hydroxyurea use, and state buy‐in in the model.

^g^ Adjusted for age at diagnosis and Yost index in the model.

## 4 DISCUSSION

This is the first population‐based cohort study to assess the potential association between statin use, overall survival, and risk of thrombosis among patients with PV and ET. We report the findings from 4010 older adults with PV (*n* = 1809) and ET (*n* = 2201) who represent the real‐world population of patients with MPN with median follow‐up of 3.92 years. We found that among patients with PV and ET, the use of statins improved survival and decreased risk of thrombosis after MPN diagnosis. The survival benefit was robust, with similar results from the primary analysis and the sensitivity analysis after excluding patients who received statins before MPN diagnosis. These findings may help guide clinical decision making regarding the use of statins in patient with MPNs.

Despite an increasing literature on the role of statins in cancer survival, there have been only a few studies evaluating statin use in patients with hematologic malignancies. In one multicenter, population‐based cohort study of patients with newly diagnosed multiple myeloma, statin use was associated with a more than 20% reduction in all‐cause mortality and myeloma‐specific mortality.^21^ However, a meta‐analysis did not support any significant impact of statins on the survival of patients with non‐Hodgkin lymphoma.^37^ Recent analyses from a prospective Canadian registry of patients with myelodysplastic syndromes showed no survival advantage in patients who received statins when compared with those who did not.^38^ Another recently published study applied methodology similar to ours and used the SEER‐Medicare database to evaluate a population of patients with myelodysplastic syndrome. PSM analysis showed improved survival and decreased progression to acute myeloid leukemia among statin users.^39^ Our study of MPN patients showed that statin use was associated with better survival as it reduced the risk of all‐cause mortality by about 22%.

A single‐center retrospective French study observed no significant association between the use of statins and the risk of thrombosis in high‐risk patients with PV and ET with no prior history of thrombosis or atrial fibrillation after diagnosis, but this study only included 192 patients from one French hospital and evaluated them for arterial or venous thrombotic events within 2 years following MPN diagnosis.^40^ Our analysis, which included a much larger number of patients with high‐risk PV and ET and a longer follow‐up of up to 12 years, demonstrated a significant risk reduction for thrombotic events of about 40% among statin users. The French study did not evaluate survival as an outcome. High incidence of thrombotic events in our study population of about 50% with 80% of them being arterial thromboses can be explained by the older patient population (median age of 77, IQR: 72–83 years), and high prevalence of cardiovascular risk factors in addition to risks intrinsic to a diagnosis of MPN.

The survival advantage among statin users in our study may in part be explained by reduced incidence of thrombotic events. There are a number of other plausible biological explanations including findings based on laboratory models. Using *JAK2‐V617F*‐dependent MPN cell lines as well as primary cells from *JAK2‐V617F* positive MPN patients, Griner et al. found that cholesterol is required for *JAK2‐V617F*‐mediated signaling and that *JAK2‐V617F*‐mediated transformation is sensitive to statins, suggesting that statins could potentially be incorporated into a therapeutic strategy for MPN patients.^41^ In addition to the potential direct effects on MPN cells, statins may also contribute to the amelioration of disease through their anti‐inflammatory effects. Chronic inflammation has been suggested as a potential trigger for the development and progression of MPNs.^27^, ^28^, ^29^ Several proposed mechanisms for the generation of chronic inflammation in MPN include an increase in reactive oxygen species generated by *JAK2‐V617F* mutated cells,^42^ increased levels of inflammatory cytokines (interleukin 1‐alpha, tumor necrosis factor‐alpha)^43^ and inflammatory gene dysregulation.^44^ Treatment with statins may lower the expression of pro‐inflammatory tumor necrosis factor‐alpha which was shown to facilitate clonal expansion of *JAK2‐V617F* positive myeloid cells of MPN patients.^45^ In addition, patients with MPN have an increased risk of developing lymphoid and solid second malignancies, which may be related to the state of chronic inflammation associated with MPN.^3^, ^46^, ^47^ Therefore, patients with MPN may potentially benefit from a number of statin‐related anti‐inflammatory and anti‐cancer effects, and additional mechanisms of action beyond antithrombotic effects may explain the survival benefit of these drugs in MPN.^14^ As the potential mechanisms underlying a possible link between statins and the outcomes among patients with MPNs are multifaceted and remain elusive, additional studies are warranted.

Based on evidence that statins reduce cardiovascular morbidity and mortality, they are recommended not only for secondary but also for primary prevention of cardiovascular events among many patients with cardiovascular risk factors including hyperlipidemia, diabetes mellitus, hypertension, and smoking (assuming an estimated 10‐year cardiovascular disease risk of 7.5%–10% or greater).^48^, ^49^ A high proportion of patients with MPN have at least one cardiovascular risk factor, including 30% of patients regardless of age and 69% of patients aged ≥60 years.^40^, ^50^ In our cohort, the percentage of patients who had hyperlipidemia, hypertension or diabetes before MPN diagnosis was even higher (80.9%, 3243 out of 4010 patients). Among the 3243 patients with cardiovascular risk factors, 37.5% (*n* = 1217) of patients did not receive statins before or after MPN diagnosis. These findings suggest a clinically‐relevant underutilization of this class of medications by older patients with MPNs.

A major strength of our study is its use of a large, population‐based cohort of older (i.e., high‐risk) patients with PV and ET treated in the real‐world setting. The nationwide Medicare claims data provided detailed information on the treatments received by patients. Furthermore, the linked SEER‐Medicare database gave us an opportunity to control for a number of other factors with potential to impact treatment decisions and risk of thrombotic events after PV/ET diagnosis, such as sociodemographic factors, comorbidity, and disability status, all of which were adjusted for in our analyses.

While our study generated important findings, there are limitations. First, medications that are not covered by Medicare, such as aspirin, could not be captured as we only used Medicare claims to obtain information about MPN management. Second, the SEER‐Medicare database did not contain information on some behavior characteristics (e.g., smoking) or results of lab tests, such as driver mutation status including *JAK2‐V617F* mutation and lipid profile to identify patients with dyslipidemia, so we could not incorporate these data into the analysis. In addition, our study is observational in design and may be subject to potential selection bias related to unobserved factors that may affect treatment decisions and outcomes of interest. However, our analysis included extensive controls for health status, prior thrombosis, sociodemographic factors, and receipt of preventive health care (influenza vaccination), which should help reduce the possibility of bias. To address immortal time bias and the possible influence on outcomes of statin use prior to MPN diagnosis, we excluded patients who initiated statins more than 1 year after MPN diagnosis from our primary analysis and limited the patient population to new statin users after MPN diagnosis in the sensitivity analysis, respectively, with sensitivity analysis showing similar results to those from primary analysis.

Overall, our study demonstrated that statins improved survival and decreased the incidence of thrombotic events in older patients with PV and ET. This novel finding supports consensus MPN clinical guidelines recommendation to address hyperlipidemia as one of the modifiable cardiovascular risk factors in patients with PV and ET and may help facilitate clinical decision making regarding the use of statins in patient with MPNs. The use of statins for patients with MPNs in the current era of ruxolitinib may have additional relevance, given that hypercholesterolemia may develop or worsen as the result of ruxolitinib use. Understanding our study limitations and taking into consideration that a randomized controlled trial of statins for patients with MPN is unlikely to be conducted, we believe that based on our results the recommendation can be made for hematologists taking care of patients with PV and ET to either be directly involved in or advocate for prescribing statins to these patients who are at a high risk for cardiovascular events.

## AUTHOR CONTRIBUTIONS

**Nikolai A. Podoltsev:** Conceptualization (equal); formal analysis (equal); funding acquisition (lead); investigation (equal); writing – original draft (lead); writing – review and editing (lead). **Rong Wang:** Conceptualization (equal); formal analysis (lead); investigation (equal); methodology (equal); writing – original draft (equal); writing – review and editing (equal). **Rory M. Shallis:** Conceptualization (supporting); investigation (supporting); writing – review and editing (equal). **Jessica M. Stempel:** Investigation (supporting); writing – review and editing (equal). **Mengyang Di:** Investigation (supporting); writing – review and editing (equal). **Natalia Neparidze:** Investigation (supporting); writing – review and editing (equal). **Amer M. Zeidan:** Conceptualization (supporting); investigation (supporting); writing – review and editing (equal). **Scott F. Huntington:** Investigation (supporting); methodology (supporting); writing – review and editing (equal). **Smith Giri:** Investigation (supporting); writing – review and editing (equal). **Sarah C. Hull:** Writing – review and editing (supporting). **Steven D. Gore:** Conceptualization (supporting); investigation (supporting); writing – review and editing (supporting). **Xiaomei Ma:** Conceptualization (equal); investigation (equal); methodology (lead); writing – original draft (equal); writing – review and editing (equal).

## FUNDING INFORMATION

This research was supported by the Frederick A. Deluca Foundation. The collection of cancer incidence data used in this study was supported by the California Department of Public Health as part of the statewide cancer reporting program mandated by California Health and Safety Code Section 103885; the National Cancer Institute's Surveillance, Epidemiology and End Results Program under contract HHSN261201000140C awarded to the Cancer Prevention Institute of California, contract HHSN261201000035C awarded to the University of Southern California, and contract HHSN261201000034C awarded to the Public Health Institute; and the Centers for Disease Control and Prevention's National Program of Cancer Registries, under agreement # U58DP003862–01 awarded to the California Department of Public Health. The ideas and opinions expressed herein are those of the author(s) and endorsement by the State of California Department of Public Health, the National Cancer Institute, and the Centers for Disease Control and Prevention or their Contractors and Subcontractors is not intended nor should be inferred. The authors acknowledge the efforts of the National Cancer Institute; the Office of Research, Development and Information, CMS; Information Management Services (IMS), Inc.; and the Surveillance, Epidemiology, and End Results (SEER) Program tumor registries in the creation of the SEER‐Medicare database.

## CONFLICT OF INTEREST STATEMENT

The authors do not have directly relevant or related conflicts to the work described in the manuscript.

## Data Availability

The datasets used to conduct this study are available upon approval of a research protocol from the National Cancer Institute. Instructions for obtaining these data are available at https://healthcaredelivery.cancer.gov/seermedicare/obtain/
